# Whole-Body Synergy-Based Balance Control for Quadruped Robots with Manipulators on Sloped Terrains

**DOI:** 10.34133/cbsystems.0201

**Published:** 2024-12-27

**Authors:** Ru Kang, Huifeng Ning, Fei Meng, Zewen He

**Affiliations:** ^1^ School of Mechanical and Electrical Engineering, Lanzhou University of Technology, Lanzhou 730050, China.; ^2^ Intelligent Robotics Institute, School of Mechatronical Engineering, Beijing Institute of Technology, Beijing 100081, China.; ^3^ Department of Mechanical Engineering, University of Tokyo, Tokyo 113-8656, Japan.

## Abstract

A quadruped robot with a manipulator that combines dynamic motion and manipulation capabilities will greatly expand its application scenarios. However, the addition of the manipulator raises the center of mass of the quadruped robot, increasing complexity in motion control and posing new challenges for maintaining balance on sloped terrains. To address this, a balance control method based on whole-body synergy is proposed in this study, emphasizing adaptive adjustment of the robot system’s overall balance through effective utilization of the manipulator’s active motion. By establishing a mapping relationship between the manipulator and the robot’s attitude angle under system equilibrium, the desired manipulator motion is guided by real-time estimates of terrain angles during motion, enhancing motion efficiency while ensuring robot balance. Furthermore, to enhance motion tracking accuracy, the optimization of system angular momentum and manipulator manipulability is incorporated into hierarchical optimization tasks, improving manipulator controllability and overall system performance. Simulation and experimental results demonstrate that the quadruped robot with a manipulator exhibits reduced velocity and attitude angle fluctuations, as well as smoother foot-end force dynamics during climbing motions with the addition of manipulator adaptive adjustment. These results validate the effectiveness and superiority of the manipulator-based adaptive adjustment strategy proposed in this paper.

## Introduction

Due to its distinctive bionic structure and motion mechanism, the bionic legged-foot robot demonstrates discontinuous, multisupport motion characteristics, facilitating efficient traversal of sloped terrains with notable throughput and agility. Integrating an additional manipulator into the quadruped robot would substantially enhance its dynamic capabilities for human-centric applications. This augmentation would empower the robot system to perform tasks like object transportation, grasping, and manipulation, thereby widening the scope of potential application scenarios for quadruped robots. Some scholars have begun to explore the application and control methods of robots in exploring space and how space robotics is evolving to meet the demands of modern space exploration and assembly missions. They emphasize innovation in robotic design, operational autonomy, and adaptability to address current challenges and expand mission capabilities [1–4]. In recent years, several research teams have delved into studies on quadruped robots equipped with manipulators, with notable examples including BigDog [5] and SpotMini [6] from Boston Dynamics, ETH’s ANYmal [7], and IIT-INAIL from HyQ [8]. Other robotics teams have also pursued related research studies on quadruped robots equipped with a manipulator [9–11].

Some latest works on using neural networks for robot arm or robot body control are potentially applicable to the investigated problem. A novel model-free dual neural network was proposed, which is able to address the learning and control of manipulators simultaneously in a unified framework [12]. Furthermore, a topology of a recurrent neural network based on a metaheuristic optimization algorithm for the tracking control of mobile-manipulator was proposed, and the proposed algorithm uses a nature-inspired optimization approach to directly solve the nonlinear optimization problem without any further transformation [13]. Current research studies about quadruped robots are concentrated on improving both motion stability and dynamic motion capabilities. Numerous studies have demonstrated these robots’ impressive adaptability to complex environments and their resilience against disturbances [14,15]. However, the incorporation of a manipulator has introduced new challenges in achieving stable control for quadruped robots navigating intricate surroundings. This arises primarily from the increased degrees of freedom (DoFs), additional weight, and heightened complexity in motion control demanded by quadruped robots equipped with a manipulator.

During the DARPA Robotics Challenge and its early stages, researchers explored the motion control of centaur robots equipped with dual manipulators. Some studies address critical challenges in dynamics, control, and path optimization for space systems, which provide robust solutions for improving accuracy, stability, and energy efficiency in operations involving robotic manipulators, tethered systems, and flexible satellites. These advancements are integral for achieving higher precision and autonomy in space missions [16–19]. Diftler et al. [20] proposed a vision-based motion planning algorithm, complemented by an embedded real-time controller, to establish a multilevel controller structure capable of efficiently managing a multitude of controllable joints. Employing an impedance-based control system on a Robonaut2 robot facilitated robust environmental interaction, alongside the attainment of precise motion and force control objectives [21]. Hebert et al. [22] integrated both move-replicating motion planning and behavioral planning techniques, which maintain stability during robot movement while achieving desired body and end-effector poses. Schwarz and Behnk’s approach [23] emphasized static stability by ensuring that all 3 limbs remain in contact with the ground, even during manipulations or grasping tasks facilitated by a fourth hand. However, this strategy poses challenges for walking concurrently with manipulations or grasping activities. Existing studies primarily focus on coordinating platform movement with upper limb manipulation or single leg–foot platform movement. The majority of approaches prioritize quasistatic, low-speed movements or primarily forward displacement through foot-end wheeling, which are constrained in their ability to control robots in unstructured, sloped terrain or during rapid movement.

In contrast, dynamic control methods address both the contact position and the force of the end effector, enabling tracking of planned target trajectories while maintaining system stability. These strategies have been utilized for constructing robust traversal of complex, unstructured terrains [24,25] and for physics-based manipulation during walking [26]. Numerous researchers have undertaken investigations into quadruped robots outfitted with manipulators characterized primarily by point-footed attributes. These studies typically involve the development of a unified dynamics model aimed at determining the target position. This model integrates an intricate system comprising a legged mobile platform and a manipulator. A simplified rigid-body dynamics model, resembling the BigDog robot equipped with a manipulator, was adopted, abstracting the legs as generalized force sources acting upon finite support bases constituted by convex hulls positioned at the feet [27]. This model comprises a 6-DoF body and a 7-DoF arm, culminating in a 13-DoF system. Leveraging the body and legs of legged robots, this configuration enhances the strength, speed, and workspace of integrated manipulators, thereby facilitating the execution of dynamic manipulation tasks. An integrated control framework was proposed, incorporating mobile platform controllers, robust model-free arm controllers, and payload estimation modules [28].

The mobile platform controller is responsible for tracking the center-of-mass (CoM) position and optimizing torso orientation while also compensating for imbalanced loads from the arms through optimization of ground reaction forces. Tournois et al. [29] introduced 2 distinct recursive methods for online identification of torso parameters, demonstrating their ability to converge toward ground-truth CoM values with high accuracy, even across rugged terrains. This innovative approach offers valuable theoretical insights into parameter identification within complex systems, such as those involving load carrying or manipulators. Bellicoso et al. [7] proposed ALMA, a motion planning and control framework tailored specifically for quadruped robots equipped with a 6-DoF manipulator. To ensure robust and reactive motions during manipulation tasks, a zero-moment-point-based online motion planning framework was utilized, alongside the generation of joint torque references by a comprehensive whole-body controller that considers the dynamics of the entire system. Sleiman et al. [30] presented a comprehensive model predictive control framework that integrates dynamic motion and manipulation tasks to plan whole-body motion trajectories. The underlying multi contact optimal control problem is formulated as a constrained switching system and solved in real time using a specialized sequential linear quadratic algorithm.

In summary, both BigDog [27] and HyQ [28] utilize hydraulic mechanisms to drive quadruped robotic platforms with a high load capacity and end-to-end manipulation capabilities. They simplify the control complexity by integrating a 6-DoF floating base system with a manipulator that corresponds to the number of DoFs. ANYmal’s quadruped robot with a manipulator achieves stable cooperation between upper and lower limbs, as well as collaborative work with humans, through the implementation of a whole-body controller considering the dynamic motion of the entire system [7], along with an overall model predictive control framework that plans motion/force trajectories for both dynamic motion and manipulation tasks of the entire body [30]. However, existing studies mainly focus on cooperative operation and stability control of quadruped robots with a manipulator on flat surfaces. Apart from SpotMini [6], which has not disclosed its control method or realization path, there are few studies addressing stability issues in complex environments for a quadruped robot with a manipulator.

To enhance stability during locomotion on a sloped terrain, this paper introduces a balance control method based on whole-body cooperative movement for quadruped robots equipped with a manipulator. This approach effectively integrates the active motion capabilities of the manipulator with whole-body cooperative control to regulate system balance. The key contributions of this study are outlined as follows:

• A balance control method grounded in whole-body synergy is proposed, emphasizing adaptive adjustment of the robot system’s overall balance through effective utilization of the manipulator’s active motion. By establishing a mapping relationship between the manipulator and the robot’s attitude angle under system equilibrium, desired manipulator motions are guided by real-time estimates of terrain angles during uphill maneuvers, enhancing motion efficiency while maintaining robot balance;
• To achieve precise motion tracking, optimization of system angular momentum and manipulator manipulability is incorporated into hierarchical optimization tasks, enhancing manipulator controllability and overall system performance.

This paper is organized as follows: Methods provides a comprehensive introduction to the system. In the “Motion tracking based on whole-body control” section, the motion tracking method based on whole-body control is described. The “Balance control of the quadruped robot in a slope” section introduces the balanced control strategy that capitalizes on the synergy between different components of the whole body, aiming to achieve enhanced stability and performance. The design and execution of simulations and experiments are meticulously detailed in Results. The last section summarizes the main contributions and findings of this research endeavor and proposes avenues for future work.

## Methods

### Quadruped robot with a manipulator

**Fig. 1.**
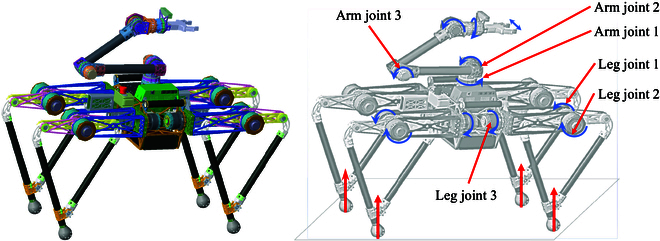
Quadruped robot with a manipulator. Each leg consists of 3 joints, the manipulator has 6 active joints, and the 3 joints near the robot torso are of particular concern in this study.

In order to achieve cooperative explosive motion and enhance impact resistance for a legged quadruped robot with a manipulator, our previous study [9,31] conducted an analysis comparing different leg structures in terms of workspace, load capacity, dynamic explosive capacity, and motion performance. The symmetrical structure leg type was selected to configure the quadruped robot with a manipulator. As depicted in Fig. 1, the total mass of the robot (including sensors, battery, and controllers) is approximately 35 kg. Each leg consists of 3 joints, with the weight primarily concentrated in the upper portion. The leg bar contributes to less than 20% of the total mass of the robot. The torso stands at a height of 80 cm, while the length of the unfolded leg results in a torso length of 120 cm. Considering joint torque requirements and motion speed, we opted for self-developed geared motors that demonstrate exceptional performance within the specified parameters. Additionally, a planetary reducer with a reduction ratio of 17:1 was chosen to strike a balance between output torque and transparency during load movement. Following torque characteristic calibration, the joints exhibit precise torque control and improved reverse drive performance.

The manipulator design was specifically tailored for symmetrical-legged quadruped robots, with the torso positioned at the system’s center [27,32]. The manipulator can be mounted in 3 locations: directly in front of the torso, behind the torso, or on the upper surface. However, due to the layout of the leg bars in the length direction of the symmetrical leg, the operating space for the manipulator attached to the front and rear surfaces of the robot torso is severely limited. To address this issue, the base of the manipulator is mounted at the center of the upper surface of the robot torso, distributing the weight evenly among all 4 legs and reducing the risk of tipping caused by an offset assembly. Mounting the manipulator on top of the robot provides a larger operating space for object manipulation and movement on a tabletop task. The manipulator can extend along the width of the body, enabling it to grasp objects on the ground while the robot maintains its standing height.

The configuration resembles a backward elbow, with the end-gripper module adjusted to the upper part of the base (coinciding with the center of the torso) to ensure the overall system’s self-balance during normal walking. Additionally, the end gripper can rotate through the shoulder yaw joint, allowing for full accessibility across the body. Through our self-developed quadruped robot with a manipulator, we have successfully achieved trotting, bounding, and pronking gaits, demonstrating robust dynamic movement capabilities.

**Fig. 2.**
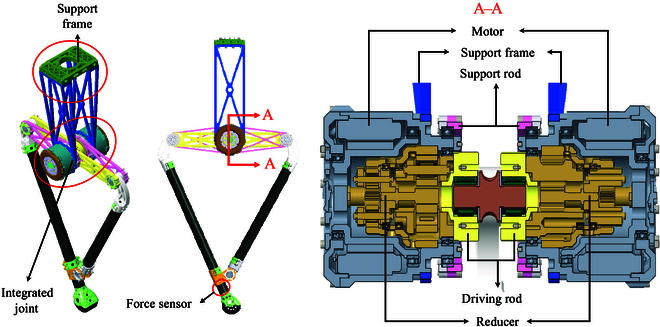
The symmetrical structure leg. As shown in the left image, the symmetrical leg is primarily composed of U-shaped supports, integrated joints, and corresponding leg rods. To elucidate the spatial layout and connection method of the integrated joints more clearly, we have sectioned the front view shown in the middle diagram along the A–A direction.

As shown in Fig. 2, 2 drive modules, each integrating a motor and a reducer, are installed on both sides of the main support base, and they are both coaxial and symmetrical. The volume and weight of the entire integrated joint module are significantly reduced while achieving uniform transmission and diffusion of impact force, thus improving the shock resistance performance of the leg structure. Moreover, as depicted in the middle diagram, the force sensor is installed at the end of the linkage near the foot sole, enabling accurate acquisition of force exertion at the foot end.

### Generalized coordinates and modeling

The quadruped robot with a manipulator is a complex multilink system characterized by high coupling, with 4 parallel legs connected in series with the manipulator. This configuration necessitates extensive calculations for dynamic modeling and optimization. To simplify the computational process, we made several assumptions based on the aforementioned analysis. Firstly, we observed that the leg mass of the robot is relatively small. Therefore, for ease of calculation, we simplified the robot as a single floating rigid body, excluding the manipulator. The robotic system is connected to the inertial coordinate system through virtual 6-dimensional forces. Consequently, the entire system can be represented as a floating 6-link structure, comprising 6 virtual driving joints connected to the inertial system (3 translational DoFs and 3 rotational DoFs), in addition to the 5 active joints from the manipulator. Notably, we did not consider the gripper’s DoF in this simplification. These simplifications facilitate the computational analysis of the quadruped robot with a manipulator, allowing us to focus on specific aspects without introducing unnecessary complexity.

The motion of the whole robot system can be described in a fixed inertial frame I. The position of the torso and the orientation of the base are denoted as $r_{\mathrm{IB}}\in {\mathbb{R}}^{3}$ and $q_{\mathrm{IB}}\in \mathrm{SO}\left(3\right)$, respectively. The joint angles of the manipulator are stacked in the vector $q_{j}\in {\mathbb{R}}^{n_{j}}$, where $n_{j}=5$. Then, the system generalized coordinate vector q and the generalized velocity vector $\overset{\cdot }{q}$ can be expressed as follows:

$$q=\left[\begin{array}{c} r_{\mathrm{IB}}\\ q_{\mathrm{IB}}\\ q_{j} \end{array}\right]\in \mathrm{SE}\left(3\right)\times {\mathbb{R}}^{n_{j}},\;\overset{\cdot }{q}=\left[\begin{array}{c} v_{\mathrm{IB}}\\ \omega_{\mathrm{IB}}\\ {\overset{\cdot }{q}}_{j} \end{array}\right]\in {\mathbb{R}}^{n_{u}}$$ (1)

where $n_{u}=6+n_{j}$ and the linear and angular velocities of the base are represented by $v_{\mathrm{IB}}$ and $\omega_{\mathrm{IB}}$_,_ respectively. The motion equation for the floating base system interacting with the environment can be expressed as

$$\left[M\left(q\right)\;-S\right]\left[\begin{array}{c} \ddot{q}\\ T \end{array}\right]+h\left(q,\;\overset{\cdot }{q}\right)=0$$ (2)

$$T={\left[F_{s}^{3\times 1}\;M_{s}^{3\times 1}\;\tau_{j}^{5\times 1}\right]}^{T}$$ (3)

where $M\left(q\right)\in {\mathbb{R}}^{n_{u}\times n_{u}}$ is the inertia matrix; $h(q,\overset{\cdot }{q})\in {\mathbb{R}}^{n_{u}}$ is the sum of gravity, centrifugal, and Coriolis forces; S is a selection matrix that selects which joints are actuated; and $F_{s}$ and $M_{s}$ represent the virtual 3-dimensional force and 3-dimensional moment of system from the ground, respectively. $\tau_{j}^{5\times 1}$ denotes the joint torque of the manipulator.

### Motion tracking based on whole-body control

**Fig. 3.**
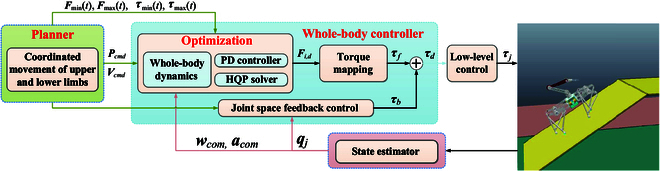
The motion control framework based on whole-body controller. In the Optimization box, PD stands for proportional-derivative and HQP stands for hierarchical quadratic programming.

As depicted in Fig. 3, the motion control framework based on whole-body control consists of several key components: a motion planner, whole-body controller, state estimator, and low-level torque controller. The following paragraphs provide a detailed explanation of each component’s role within the framework.

The motion planner plays a crucial role in determining the appropriate gait type based on the desired motion goal. It then proceeds to plan the position, velocity, acceleration, body angle, angular velocity, and angular acceleration of CoM motion. This comprehensive planning process ensures efficient and coordinated movement of the robot. The whole-body controller primarily focuses on achieving the target values for the body position and angle as planned by the user. By considering the constraints imposed by the whole-body dynamics model, friction cone, and joint driving capabilities, it utilizes a hierarchical optimization method to determine the optimal contact forces during each control cycle iteration. Subsequently, these optimal solutions are mapped to the joint space and transmitted to the low-level torque controller. To ensure accurate feedback and reliable information for the upper-level planning and whole-body control processes, the state estimation module combines data from attitude sensors, force sensors, and joint encoders. By incorporating the kinematic model of the leg, this fusion of information allows for the calculation of the CoM position, attitude, contact state, and ground inclination. These estimations contribute to robust and precise feedback information, enhancing the effectiveness of the overall control system. Overall, the motion control framework based on whole-body control integrates multiple components that work in tandem to enable sophisticated and coordinated robotic motion.

### Hierarchical optimization

The prioritization of tasks is a critical aspect of robot motion control, as it ensures accurate motion tracking while optimizing system efficiency. Traditional quadratic programming (QP) approaches achieve target motion optimization by assigning weight coefficients based on task priorities. However, achieving absolute priority among different tasks using this method can be challenging. To address this issue, we propose an approach that adopts hierarchical optimization to solve a series of QP problems by progressively reducing the solution space based on task priorities. One or more tasks with the same priority, denoted as *W*, can be defined through a set of linear equations and inequality constraints on the solution vector **X**:

$$W:\left\{\begin{array}{c} A_{\mathrm{eq}}X-b_{\mathrm{eq}}=\varepsilon \\ A_{\mathrm{ieq}}X-b_{\mathrm{ieq}}⩽v \end{array}\right.$$ (4)

where X is the optimization objective and ε and v are the relaxation variables to be minimized. $A_{\mathrm{eq}}$, $b_{\mathrm{eq}}$, $A_{\mathrm{ieq}}$, and $b_{\mathrm{ieq}}$ represent the coefficient matrices of equality constraints and inequality constraints, respectively.

By solving these tasks in a specified priority order, an optimal solution $X^{*}$ can be obtained. Previous studies have explored specific iterative optimization methods for this purpose [9].

### Formulation of tasks to be optimized

In the context of the motion framework depicted in Fig. 3, we begin by defining the motion trajectory in the operational space for specific system components, such as the manipulator and torso, within the advanced controller. This trajectory includes parameters like torso position, base direction, and manipulator position and attitude. To generate the trajectory, we employ the cubic spline interpolation method, which ensures that starting and ending velocities and accelerations are equal to zero.

Subsequently, the expected force is optimized using the whole-body controller. We formulate the control problem as a QP problem comprising linear equality and inequality tasks. To solve this optimization problem, we utilize a hierarchical optimization algorithm. Referring to Eqs. 2 and 3, the optimization objective for the simplified 6-link model can be uniformly expressed as

$$X={\left[\begin{array}{cc} {\ddot{q}}^{T} & T^{T} \end{array}\right]}^{T}$$ (5)

Firstly, it is essential to incorporate inequality constraints, such as the joint torque limit and the contact force limit resulting from the friction cone:

$$\left\{\begin{array}{c} \tau_{\text{min}}\leq \tau \leq \tau_{\text{max}}\\ -{\mathrm{uF}}_{z}\leq F_{x/y}\leq {\mathrm{uF}}_{z} \end{array}\right.$$ (6)

where *u* is the friction coefficient of the contact surface.

A crucial aspect of motion tracking is defining appropriate constraints based on the desired motion. In the context of whole-body synergistic motion, this paper focuses on task constraints that account for these motion characteristics. These task constraints primarily include the following:

• *System dynamics model.* The dynamic motion of the robot must adhere to the system dynamics model. This constraint is imposed as an equation and holds the highest priority. The corresponding QP standardized model matrices are as follows:

$$\left\{\begin{array}{c} A_{\mathrm{eq}}=\left[\begin{array}{cc} M\left(q\right) & -S \end{array}\right]\\ b_{\mathrm{eq}}=h(q,\overset{\cdot }{q}) \end{array}\right.$$ (7)

• *Contact constraint.* Ensuring that the support point remains in contact with the ground without sliding is a critical condition during the support phase of legged robots. This objective can be achieved by constraining the target acceleration:
  $$I_{22}X=\left[\begin{array}{c} {\ddot{q}}_{\text{torso}\_d}^{6\times 1}\\ 0_{16\times 1} \end{array}\right]$$ (8)
  where ${\ddot{q}}_{\text{torso}\_d}^{6\times 1}$ is the desired acceleration vector of the torso obtained from the planner.

• *Angular momentum task tracking.* In order to ensure system stability in dynamic motion, the angular momentum task tracking of the robot can be defined as
  $$\left[\begin{array}{cc} J_{H} & 0_{11} \end{array}\right]X={\overset{\cdot }{H}}^{d}-{\overset{\cdot }{J}}_{H}\overset{\cdot }{q}$$ (9)
  where $\overset{\cdot }{H}$ and $J_{H}$ represent the angular momentum based on separation angular acceleration and the center momentum matrix, respectively.

• *Motion tracking in operational space.* The control objective is to achieve accurate motion tracking and ensure that the system moves along the desired trajectory. In the context of jumping motion planning, it is crucial to coordinate the motion of both the upper and lower limbs simultaneously. By referring to the pre-planned trajectories of the torso and manipulator shown in Fig. 3, motion tracking in the operational space can be realized by imposing the following constraints:

$$\begin{array}{c} \left[\begin{array}{ccc} I_{6} & J_{\mathrm{arm}}^{5\times 5} & I_{11} \end{array}\right]X=\\ \left[\begin{array}{c} {\ddot{q}}_{\text{torso}\_d,p}^{3\times 1}+K_{p,p}\left(p_{\text{torso}\_d}-p_{\text{torso}}\right)+K_{d,p}\left({\overset{\cdot }{p}}_{\text{torso}\_d}-{\overset{\cdot }{p}}_{\text{torso}}\right)\\ {\ddot{q}}_{\text{torso}\_d,\omega }^{3\times 1}+K_{p,\omega }\text{log}\left(R_{d}R^{T}\right)+K_{d,\omega }\left(\omega_{\text{torso}\_d}-\omega_{\text{torso}}\right)\\ {\ddot{q}}_{\mathrm{arm}\_d}^{5\times 1}-{\overset{\cdot }{J}}_{\mathrm{arm}}^{5\times 5}{\overset{\cdot }{q}}_{\mathrm{arm}}\\ 0_{11} \end{array}\right] \end{array}$$ (10)

where $J_{\mathrm{arm}}^{5\times 5}$ denotes the Jacobian of arm-end motion and the desired acceleration $\ddot{q}$ is obtained using a proportional-derivative control law and can be divided into 3 parts: torso position, base orientation, and manipulator’s position. The orientation error is determined using the exponential map representation of rotations; $R_{d}$ and *R* represent the desired and actual base orientations, respectively.

• *Manipulability optimization of the manipulator.* For the quadruped robot with a manipulator discussed in this paper, accurate motion tracking is crucial. Additionally, certain motion directions require high joint rates and forces when the arm is in a specific configuration. It can also be challenging to move in certain directions near singularities. The manipulability of a robot refers to its ability to move freely in all directions within the workspace. In this study, we investigate local manipulability using the manipulator’s Jacobian matrix, which establishes the relationship between infinitesimal joint motions and infinitesimal workspace motions.
  The maximum singular value of the Jacobian matrix primarily indicates the ability of the drive module to withstand unknown external disturbances. A smaller value signifies better shock resistance of the manipulator [33]. The manipulation performance of the manipulator can be described by the following function:
  $$m\left(\theta_{m}\right)=\sqrt{\text{det}\left({}^{M}J_{\mathrm{arm}}^{T}{\left(\theta_{m}\right)}^{M}J_{\mathrm{arm}}\left(\theta_{m}\right)\right)}$$ (11)
  According to the relationship between the length of the large and small arms of the manipulator, it can be seen that the value of the joint $\theta_{m3}$ has the largest weight on the position of the end of the manipulator and its effect on the acceleration in the *Z* direction [34], so its effect on the acceleration in the *Z* direction of the end of the manipulator can be obtained through the mapping relationship of the joint $\theta_{m3}$ in the Jacobian matrix:
  MZ¨mdes=∂mθm∂θm3−kdMZ·m=mθmTr∂MJarm∂θm3⋅MJarm†−kdMZ·m (12)
  where $k_{d}$ denotes the coefficients of the velocity damping phase at the end of the manipulator, so this task constraint can be described as
  $$\left[\begin{array}{cc} S_{\mathrm{zm}} & 0_{11} \end{array}\right]X={\;}^{M}\;{\ddot{Z}}_{m}^{\mathrm{des}}$$ (13)
  where $S_{\mathrm{zm}}$ is the row vector that selects the corresponding component in the optimization objective *X*.
  • *Energy optimization.* Similar to the optimization goal of motion tracking, we can also incorporate the task of driving force or torque. These tasks typically have the lowest priority and are utilized to enhance the energy efficiency of the system.
  $$\left[\begin{array}{cc} 0_{11} & I_{11} \end{array}\right]X=0$$ (14)

The aforementioned task constraints are crucial considerations for legged robots in motion control. The specified order of task priority in this paper is presented in Table 1. The highest-priority task encompasses adherence to the dynamics model, physical constraints of the actuator, and friction cone constraints. The second-order priority ensures tracking of the desired motion. Lastly, the task constraints are employed to optimize the manipulator’s manipulation capability and improve energy efficiency.

**Table 1.** Descriptions of task priorities

Priority	Task
1	Equation of motion
	No contact motion
	Angular momentum tracking
2	Torso position tracking
	Torso orientation tracking
	Manipulator motion tracking
3	Manipulability optimization
	Contact force minimization
	Joint torque minimization

The simplified dynamic model of the system, as depicted in Eq. 2, has been described above. By integrating the planned trajectory and motion optimization using hierarchical optimization techniques, we obtain the optimized target variables X, which includes the 6-dimensional force acting on the torso and the torque of the 5 joints from the manipulator, and the latter can be used as the input of low-level control directly. Simultaneously, it is necessary to map the virtual 6-dimensional force ${\left[F_{s},\;M_{s}\right]}^{T}$ applied to the torso in the operational space to the joint space of the robot’s legs. Specific iterative optimization methods for achieving this mapping have been documented in previous studies [9].

### Balance control of the quadruped robot in a slope

In order to achieve stable motion on a sloped terrain, it becomes necessary to dynamically adjust the robot’s attitude and motion planning based on real-time estimated terrain information. This section introduces control strategies for robotic systems to adapt to such challenging terrains. Specifically, we focus on the analysis of attitude adjustment in the elevation direction as an example. On a flat terrain, the ideal equilibrium state of the robot can be described as follows: the torso *Z* axis is aligned with gravity, the *X* axis is perpendicular to gravity, and a constant or desired body height is maintained. However, when traversing sloped terrains like slopes or steps, the robot’s torso must adapt to changing support planes in order to establish a new state of equilibrium.

**Fig. 4.**
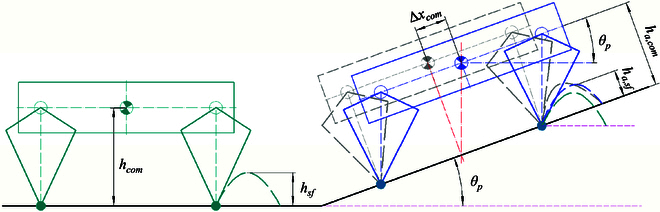
Walking diagram of a quadruped robot. The green model illustrates the equilibrium state on flat ground. The gray model represents the state achieved by rotating only the tilt angle during the transition phase, while the blue model signifies the adaptive state on a slope.

As depicted in Fig. 4, to maintain balance while the robot is on a slope, it becomes necessary to rotate the orientation of the equilibrium pose on the horizontal plane, as demonstrated in the gray model. This process solely focuses on adjusting the orientation of the torso. In accordance with the stability criterion, the combined force and moment acting on the robot must be zero. Additionally, due to gravitational perturbations in this state, the support legs (represented by virtual dashed lines of the same color) must be adjusted parallel to the direction of gravity.

In addition, this adjustment of the torso orientation results in a shift in the position of the CoM by an offset $\Delta x_{\mathrm{com}}$ in the upward direction of the slope. Consequently, the leg length of the initial state increases accordingly. To ensure a sufficient range of adjustability for the leg during future unknown motions, it is essential to fine-tune the CoM position along the direction of gravity. Through simplification, we can derive the following equations that describe the adjustments in the upward and vertical directions of the slope when considering a slope with an angle $\theta_{p}$; the adjustments in the upward and vertical directions of the slope are, as shown in Eq. 15:

$$\left\{\begin{array}{c} \Delta x_{\mathrm{com}}=h_{\mathrm{com}}\times \text{sin}\;\theta_{p}\\ h_{a,\mathrm{com}}=h_{\mathrm{com}}\times \text{cos}\;\theta_{p} \end{array}\right.$$ (15)

When walking on an inclined slope, it becomes necessary to adjust the trajectory of the swinging leg to accommodate real-time changes in the support plane. As illustrated in Fig. 4, we initially rotate the planned foot trajectory on flat ground to align with the slope (from the green dashed line to the gray dashed line). However, due to the inclination of the slope, the positioning of the torso relative to the foot has changed, rendering the previous foot planning incompatible with the current state. In order to achieve real-time synchronization between the swing leg trajectory and the support plane, we adjust the corresponding swing leg trajectory based on the CoM position, as depicted in Eq. 16:

$$\left\{\begin{array}{c} \Delta x_{\mathrm{sf}}\;=h_{\mathrm{sf}}\;\times \;\text{sin}\;\theta_{p}\\ h_{a,\mathrm{sf}}\;=h_{\mathrm{sf}}\;\times \;\text{cos}\;\theta_{p} \end{array}\right.$$ (16)

In the above equation, $h_{\mathrm{sf}}$ is the highest point of swing on flat ground, $h_{a,\mathrm{sf}}$ is the adaptive highest point on slopes, and $\Delta x_{\mathrm{sf}}$ is the compensated offset of the swing leg in the forward direction.

To calculate the real-time terrain angle, we have proposed a terrain estimation method based on the generalized least squares approach in previous research [35]. In this method, foot positions in world coordinates are obtained by integrating encoder information from the inertial measurement unit to encode trunk orientation information, eliminating the need for additional perception.

### Adaptive attitude balance control based on a manipulator

Combined with the aforementioned analysis, when a quadruped robot traverses sloped terrains such as slopes and steps, it becomes necessary to adjust the body’s attitude angle in order to adapt to the inclination of the support plane and maintain balance within the robotic system. Simultaneously, adjustments to the body’s height during motion are required to ensure sufficient leg extension. As indicated by Eqs. 15 and 16, the robot’s body height and the swing height of the swinging leg will decrease as the inclination angle of the slope plane increases. This adjustment is crucial for maintaining a balanced state throughout the motion. However, it should be noted that this method may become unreliable for terrains with poor unevenness due to the limitations on the lift height of the swing leg.

**Fig. 5.**
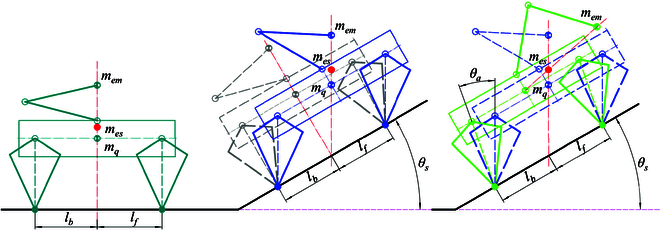
Adaptive adjustment strategy based on manipulator. The left image depicts the equilibrium posture of the quadruped robot with a manipulator on a flat surface; the solid red circle represents the equivalent center of mass (CoM) of the quadruped robot system with a manipulator.

In order to enhance the performance of a quadruped robot with a manipulator when traversing a sloped terrain, this paper presents an adjustment strategy based on manipulator adaptation derived from the robotic system’s topology. The fundamental concept behind this method is to utilize the active motion characteristics of the manipulator to effectively counterbalance a portion of the overturning moment resulting from the angle of the support plane. This approach aims to minimize the adjustments required for the support leg and swing leg, thereby improving the robot’s ability to traverse sloped terrain. As illustrated in Fig. 5, the projection of the CoM in the direction of gravity aligns with the endpoint of the support leg ($l_{b}=l_{f}$), and the weight of the system, $m_{\mathrm{es}}$_,_ is denoted as

$$m_{\mathrm{es}}=m_{\mathrm{em}}+m_{q}$$ (17)

where $m_{\mathrm{em}}$ and $m_{q}$ denote the equivalent mass of the manipulator and the quadruped robot mass, respectively.

Similar to the adjustment strategy of the quadruped robot on a slope in the previous section, a quadruped robot with a manipulator can maintain balance on a slope on the premise that the projection of the equivalent CoM of the system in the direction of gravity falls on the midpoint of the line connecting the support feet. Therefore, the manipulator needs to adjust its attitude accordingly so that the equivalent CoM of the manipulator $m_{\mathrm{em}}$ is located on the plumb line where the CoM of the quadruped robot is located, in order to ensure that the projection of the equivalent CoM of the whole system $m_{\mathrm{es}}$ in the direction of gravity coincides with that of the CoM of the quadruped robot. The robot state, as indicated by the blue line in the middle schematic of Fig. 5, is in equilibrium on an inclined slope. However, the strategy based on the adjustment of the quadruped robot’s torso posture can ensure balance when standing when the slope angle $\theta_{s}$ is large, but the step length of the swing leg and the height of the leg lifting will be reduced, thus affecting the adaptation ability on the slope surface.

Combining the above analyses, this paper proposes an adjustment strategy based on manipulator adaptation as indicated by the green line shown in the right panel of Fig. 5. The method ensures that the equivalent CoM $m_{\mathrm{es}}$ of the system is always on the plumb line where the center of the support point is located by simultaneously adjusting the position of the equivalent CoM $m_{\mathrm{em}}$ of the manipulator and the body attitude of the quadruped robot under the condition of keeping the contact state of the foot end unchanged. The position of the equivalent CoM of the system after the adjustment coincides with the position before the adjustment, as shown in the right of Fig. 5.

**Fig. 6.**
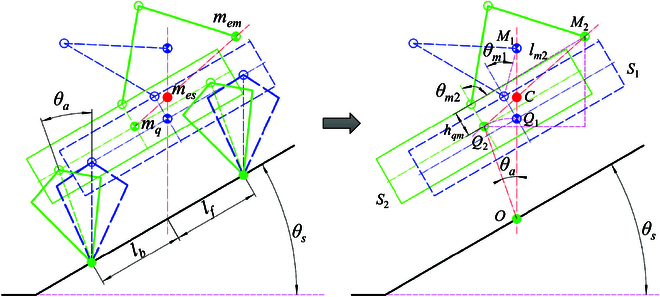
Geometric analysis of adaptive adjustment of the manipulator. The right figure represents the simplification of the support center of the robot’s 4 legs to the intersection of the plumb line and the slope surface.

The motion of the manipulator required to maintain the balance of the quadruped robot system with a manipulator mainly consists of 3 variables: the length of the manipulator $l_{m}$, the swing angle of the manipulator $\theta_{m}$, and the yaw angle of the manipulator $\theta_{y}$, through which the angular information of the manipulator’s 1, 2, and 3 joints can be computed to find out the positional information of the end of the manipulator in the 3-dimensional space. In this section, in order to illustrate the adjustment strategy based on the swing of the manipulator more clearly, the motion of the manipulator is projected into the *XOZ* plane to analyze the stable control of the robot’s attitude in the pitch direction by the adjustment of the manipulator length $l_{m}$ and the manipulator $\theta_{m}$ swing angle. We combine the robot state $S_{1}$ depicted by the blue line as shown in Fig. 6, and to minimize the height of the equivalent CoM of the manipulator, we set $l_{m1}=l_{m\;\text{min}}$. When the manipulator is deflected with the robot $\theta_{m1}=\theta_{s}$, the distance from the CoM of the manipulator to the CoM of the quadruped robot $Q_{1}M_{1}$ can be found according to the geometrical relation

$$Q_{1}M_{1}=\sqrt{{l_{m\;\text{min}}}^{2}+{h_{\mathrm{qm}}}^{2}-2l_{m\;\text{min}}h_{\mathrm{qm}}\;\text{cos}\;\left(\theta_{m1}\right)}$$ (18)

where $h_{\mathrm{qm}}$ denotes the base of the manipulator with the quadruped robot CoM displaced in the torso height direction. The height of the CoM of the robot system at equilibrium can then be calculated as

$$\mathrm{OC}=\frac{{\mathrm{OQ}}_{1}\times m_{q}+{\mathrm{OM}}_{1}\times m_{\mathrm{em}}}{m_{q}+m_{\mathrm{em}}}$$ (19)

When the contact point of the quadruped robot with the slope surface is unchanged, the position of the point *C* calculated above is the lowest equilibrium point of the system CoM. Therefore, in the process of adaptively adjusting the body attitude of the quadruped robot through the manipulator, we take the point *C* solved above as the immovable point of the CoM of the system. As shown in the right figure of Fig. 6, we set the state based on the manipulator after the adjustment as $S_{2}$; the adjustment process is given the desired quadruped robot attitude angle adjustment value $\theta_{a}$, solving the target’s manipulator length $l_{m2}$ and swing angle $\theta_{m2}$, and the solution process is as follows:

$$Q_{2}C=\sqrt{{{\mathrm{OQ}}_{2}}^{2}+{\mathrm{OC}}^{2}-2{\mathrm{OQ}}_{2}\times \mathrm{OC}\times \text{cos}\;\left(\theta_{a}\right)}$$ (20)

$$Q_{2}M_{2}=Q_{2}C\left(1+\frac{m_{q}}{m_{\mathrm{em}}}\right)$$ (21)

$$l_{m_{2}}=\sqrt{Q_{2}{M_{2}}^{2}+{h_{\mathrm{qm}}}^{2}-2Q_{2}M_{2}\times h_{\mathrm{qm}}\times \text{cos}\;\left(\theta_{x}\right)}$$ (22)

$$\theta_{x}=\frac{\pi }{2}+\theta_{s}-\text{arccos}\left(\frac{{\mathrm{OQ}}_{2}\times \;\text{sin}\;\left(\theta_{a}\right)}{Q_{2}C}\right)$$ (23)

Combining Eqs. 20 to 23, the desired swing angle of the manipulator can be obtained from the geometric relationship

$$\theta_{m2}=\pi -\text{arccos}\left(\frac{{l_{m_{2}}}^{2}+{h_{\mathrm{qm}}}^{2}-Q_{2}{M_{2}}^{2}}{2l_{m_{2}}h_{\mathrm{qm}}}\right).$$ (24)

When passing through the sloped terrain, the real-time slope information is obtained through the state estimation method, and when the slope inclination is not greater than the set safety threshold, the quadruped robot with a manipulator can adapt to the terrain completely through the body attitude angle. When the slope inclination angle is greater than the set safety threshold, the adaptive motion of the manipulator is solved by combining Eqs. 22 and 24 with the quadruped robot’s body attitude adjustment value $\theta_{a}$. The above mentioned manipulator adjustment can correct $\theta_{p}$ to $\theta_{s}-\theta_{a}$ in Eqs. 15 and 16, which can reduce the adjustment amount of the robot’s support leg and swing leg at the stage of the slope, and improve the stability and efficiency of the slope climbing.

## Results

In order to validate and further optimize the motion tracking method and adjustment strategy described above, comparative simulation experiments are set up in this section. This paper utilizes the CoppeliaSim dynamic software for motion simulation, with the physical parameters of the robot and simulation parameters during motion detailed in Table 2.

**Table 2.** Parameters for simulations and experiments

Symbol	Physical quantity	Numerical value
$M_{s}$	Total robot mass	43 kg
$M_{\mathrm{sl}}$	Single leg mass	1.4 kg
$M_{m}$	Manipulator mass	8 kg
$M_{\mathrm{me}}$	Manipulator end mass	3 kg
$L_{\text{body}}$	Robot length	1.2 m
$W_{\text{body}}$	Robot width	0.4 m
$h_{\mathrm{mm}}$	Maximum arm length	0.7 m
$h_{\mathrm{ijm}}$	Initial arm length	0.2 m
*Step*	Control cycle	1 ms
$h_{s}$	Leg lift height	0.08 m
*g*	Acceleration of gravity	9.8 m/s^2^
$K_{p}^{B}$	Robot torso stiffness coefficient	[2000,2000,2500,3000,4000,2000]
$K_{d}^{B}$	Robot torso damping coefficient	[150,100,150,120,120,100]
$K_{p}^{M}$	Robot arm joint stiffness factor	[1000,2000,2000,500,20]
$K_{d}^{M}$	Coefficient of damping of manipulator joints	[60,80,80,10,0.5]
μ	Coefficient of friction	0.7

### Simulations

When the robot navigates on a steep slope, achieving stable motion tracking solely through adjusting the robot’s body posture becomes challenging. To validate the manipulator adaptive control method proposed for a sloped terrain in this study, this section introduces upper and lower limb cooperative motion on a 30°$30^{{}^{\circ}}$ slope. Users can predefine the manipulator adaptive adjustment threshold. The manipulator adaptive adjustment initiates when the angle of the support plane surpasses this threshold, aiming to adjust the actual CoM position of the robotic system via manipulator swinging. Consequently, it adjusts the trajectories of the support leg and the swinging leg.

**Fig. 7.**
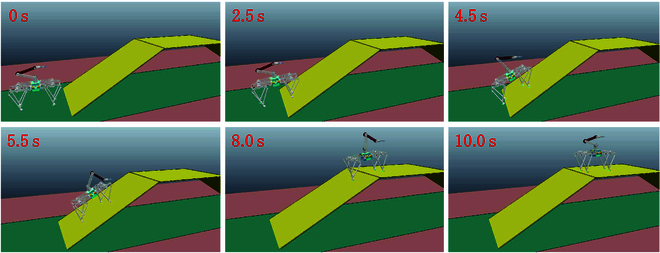
The snapshots of robot uphill movement with manipulator adaptive adjustment. The robot initiates its movement from flat ground and transitions onto the preset 30° slope and reaches the top flat plane after traversing the slope.

Figure 7 shows the manipulator adaptive adjustment process on a 30° slope. Once the angle of the robot’s support plane exceeds 20°, the manipulator adaptive adjustment is activated. Real-time calculations determine the swing angle and length of the manipulator based on the estimated angle of the support plane. Conversely, if the robot’s support plane angle falls below 20°, the manipulator adaptive adjustment is deactivated, and the manipulator reverts to its initial state.

**Fig. 8.**
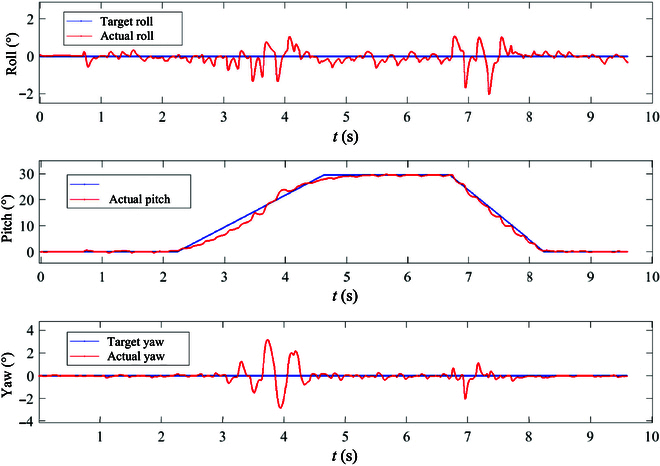
Body attitude angle variations on a 30° slope. The 3 subfigures present the tracking curves of the roll, pitch, and yaw of the robot during uphill motion, respectively.

Figure 8 illustrates the robot’s body attitude angle changes during manipulator adaptive adjustment on a 30° slope. Analyzing the robot’s pitch angle tracking curve reveals key transitions: The robot begins its slope entry at 2.2 s, transitioning from the flat ground to the 30° slope surface until 4.6 s, characterized by a gradual increase in the body pitch angle with the slope gradient. Between 4.6 and 6.8 s, the robot fully navigates the slope, demonstrating improved alignment between the body pitch angle and the slope angle. From 6.8 to 8.2 s, the robot gradually transitions back from the 30° slope to a flat surface.

The results indicate minimal tracking error on the 30° slope, with a larger pitch angle following errors noted during slope entry and exit phases. These errors stem from mismatches between the friction coefficient, damping coefficient of the robot’s CoM stiffness, and actual working conditions due to rapid changes in slope angle. This mismatch leads to minor swing leg sliding upon ground contact. Optimizing these parameters in physical robot experiments is recommended. Additionally, the robot’s roll angle fluctuated between −2° and 1°, while the yaw angle fluctuated within ±3°. Larger fluctuations occurred during shorter cycles of slope entry (3.5 to 4.6 s) and slope exit (6.8 to 8.0 s), with relatively small tracking errors observed in other phases.

**Fig. 9.**
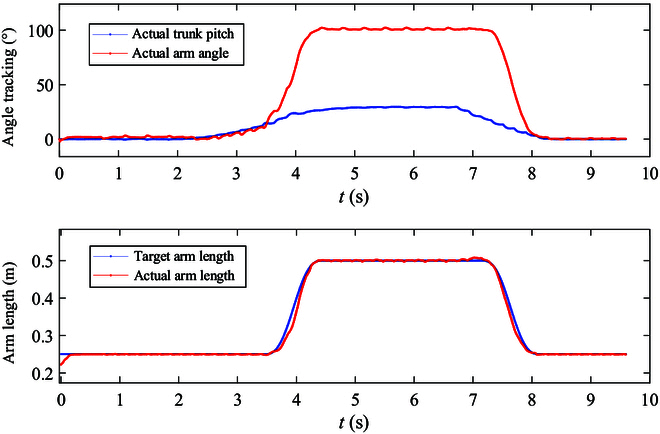
Adaptive adjustment of the manipulator on a 30° slope. To provide a clearer analysis of the motion of the manipulator during the uphill traversal, we represented the position of the manipulator end in a polar coordinate system using the angle and length of the whole manipulator. The first figure illustrates the mapping relationship between the actual manipulator angle and robot’s pitch; the second figure depicts the relationship between the actual and desired lengths of the manipulator.

In order to delve deeper into the motion tracking during the slope entry and exit phases, the variations in the manipulator’s swing angle and length throughout the uphill process are depicted in Fig. 9. The manipulator’s swing angle progression is as follows: It gradually increases from the initial angle to 20°, aligning with the robot’s torso pitch angle, reaching 20° between 2.2 and 3.5 s. Subsequently, from 3.5 to 4.6 s, the swing angle escalates to 103°, while the manipulator’s length extends to 0.5 m. Referring to the robot’s body pitch angle curve in Fig. 8, it is evident that the robot’s pitch angle changes in real-time correlation with the slope angle during this climbing phase of manipulator adaptive adjustment. This manipulation alters the motion of the robot’s support leg and swing leg by moving the manipulator while maintaining the robot’s body attitude angle constant, hence enhancing system stability margin and motion efficiency. Upon analyzing the manipulator’s adaptive control activation when the slope angle surpasses the preset threshold (20°) and its swift adjustments to accommodate slope changes from 3.5 to 4.6 s, resulting in some fluctuations in the robot’s attitude angle tracking, enhancements can be made by refining the slope threshold for adaptive adjustments and the manipulator’s motion parameters.

### Experiments

To validate the efficacy of the control method utilizing manipulator adaptation proposed in this paper, this section conducts physical comparison experiments using a quadruped robot with a manipulator climbing a 25° slope from flat ground. Two experiments are conducted: one where the manipulator remains stationary during uphill movement and the other incorporating manipulator adaptive adjustment.

The motion parameters for the uphill process are detailed in Table 2. These parameters include setting the swing leg’s lift height at 0.12 m and the ground friction coefficient at 0.4 while maintaining consistency across all other parameter settings. Considering the robot’s mass ratio and the manipulator’s end, the balance control adjustment threshold is established at 20° for the climbing experiment. When the slope angle exceeds 20°, the manipulator swings to adjust the CoM position, ensuring that the deflection angle between the robot’s torso and legs does not surpass this threshold.

**Fig. 10.**
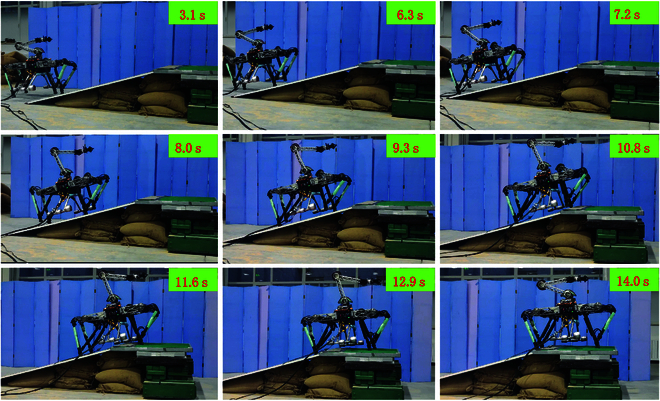
Uphill motion of the quadruped robot with a manipulator on a 25° slope. The manipulator adapts its positioning according to the actual slope angle and the pitch angle of the robot during the uphill process. (The videos of the robot uphill the slope are displayed in the Supplementary Materials.)

Figure 10 portrays a snapshot of the robot’s uphill motion process. The robot commences its slope entry from flat ground at 3.1 s, with a transition period lasting from 3.1 to 7.2 s as it gradually ascends the 25° slope. During the slope entry phase, both the pitch angle of the quadruped robot body and the manipulator’s swing angle continuously adjust in response to the slope angle, ensuring that the system maintains dynamic balance. As the slope angle surpasses the preset threshold (20° in this experiment), triggering the manipulator adaptive adjustment strategy, real-time calculations determine the necessary manipulator position to uphold system stability. Once the robotic system is fully on the slope (with a constant support plane angle), the manipulator retains its position until the support plane angle falls below the set threshold, at which point it resets.

**Fig. 11.**
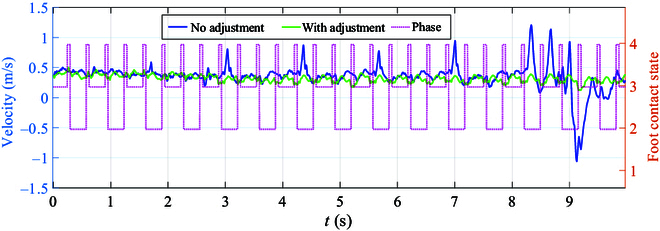
Robot velocity curve during uphill movement. This figure depicts the velocity fluctuation during the robot’s uphill traversal with and without manipulator adjustment while also providing insights into the changes in the robot’s contact states with a pink dashed line.

In order to assess the enhancement in uphill performance resulting from the inclusion of manipulator adaptation, we present the motion velocity curve of the robot’s CoM in 2 experiments, displayed in Fig. 11. The desired speed for the robot’s uphill movement is set at 0.35 m/s. Analysis of the velocity curve reveals that significant fluctuations in velocity occur frequently during motion without manipulator adjustment, indicating poor system stability. Conversely, upon implementing manipulator adaptive adjustment, the speed fluctuations in robot movement consistently remain within a narrow range. To further evaluate the uphill performance improvement following manipulator adaptive adjustment, corresponding curves depicting the foot-end force of the right rear leg and the robot’s phase during motion are provided for analysis.

**Fig. 12.**
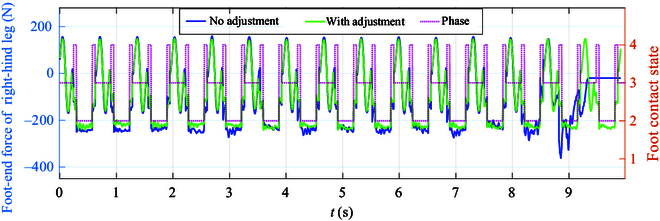
Foot-end force profile of the robot’s right hind leg during uphill movement with and without adjustment while also providing contact phase changes with a pink dashed line.

Figure 12 shows that the foot-end force of the right hind leg relative to the phase during the robot’s uphill movement illustrates regular and periodic changes throughout the entire process. With the inclusion of the manipulator adaptive adjustment strategy, notable observations can be made: The *Z*-direction foot-end force is approximately 110 N during the quadruped support phase and around 220 N in the diagonal support phase for the right hind leg, almost matching the total weight of the robotic system with a manipulator (43 kg) with minimal error. The smooth parabolic trend of the *Z*-direction force changes during the swing phase of the right hind leg validates the stability of the swing leg motion tracking. Throughout the uphill motion, the *X*-direction force exhibits a consistent periodic pattern, with slightly higher peak forces in the support phase compared to flat ground movement, reaching about −20 N. Similarly, the *Y*-direction force fluctuates between 0 and 5 N, ensuring lateral stability and preventing sideways sliding. Conversely, in motion without manipulator adjustment, significant foot-end force fluctuations occur, with a peak value of 360 N during the diagonal support phase, indicating poor stability at that juncture. This analysis highlights that the addition of the mechanical arm adjustment method maintains the robot’s lower limb in a periodic and stable motion state during uphill movement, affirming the stability and reliability of the whole-body control-based motion tracking method proposed in this study.

**Fig. 13.**
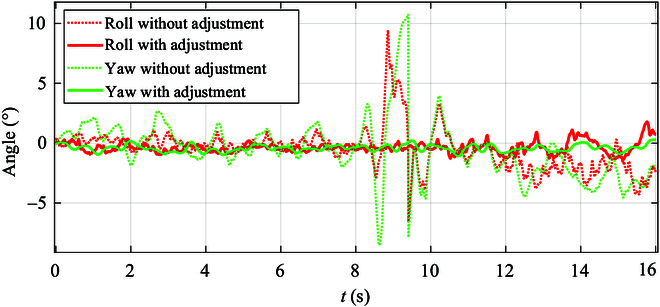
The roll and yaw variation curves of the quadruped robot during uphill movement with and without adjustment.

In order to further analyze the quadruped robot with a manipulator during uphill motion, we present the tracking error of the robot’s attitude angle throughout the movement process with manipulator adjustment, illustrated in Fig. 13. Key observations from the tracking errors include the following: The robot’s yaw angle error remains relatively small, fluctuating between −1° and 0°. The traverse angle fluctuates within the range of −1.2° to 1.8°, with the largest error occurring during the final stage of descending the slope.

From Fig. 13, it can be observed that the majority of the angle fluctuations in the roll and yaw directions of the robot range between −4.8° and 4.2° in the motions without manipulator adjustment. However, the maximum angle error exceeds 10° between the 8th and 10th seconds. This is attributed to the poor balance of the robot, leading to slipping of the foot sole when no manipulator adjustment are made. This discrepancy is attributed to limited space on the support plane at the slope’s endpoint, hindering a seamless transition from slope to horizontal ground.

**Fig. 14.**
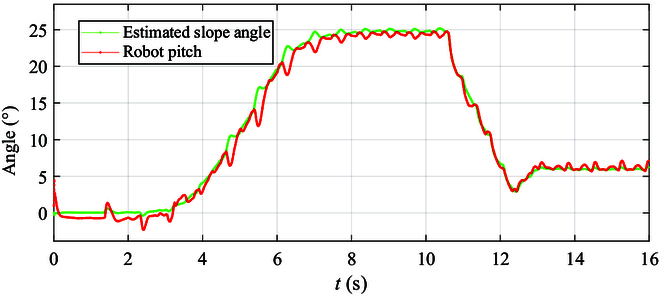
The pitch variation curves of the quadruped robot during uphill movement.

Examining the robot’s pitch angle following the curve in Fig. 14, it is evident that the robot maintains a pitch angle of around 6.5° from 13 to 16 s. The slope estimation transitions from near 0° (flat ground) at the start to gradually increasing to 25° from 3.1 to 7.2 s, stabilizing between 24° and 25° thereafter. The minimal error in the robot’s pitch angle following the slope estimation showcases real-time adaptability to slope changes, validating the effectiveness of the control strategy integrating manipulator adaptation proposed in this study.

Based on the comprehensive analysis of experimental data pertaining to the quadruped robot with a manipulator in uphill movement, the incorporation of manipulator adaptive adjustment results in improved stability during diagonal walking transitions, reduced speed fluctuations, enhanced foot-end force consistency, and minimized tracking error in the robot’s torso attitude angle. These outcomes confirm the validity of the manipulator adaptive adjustment strategy proposed in this paper. At least 2 test results indicated that the proposed method can achieve stable uphill and downhill movements for robots. To further validate the system’s reliability and repeatability, we will conduct multiple experiments on the robot’s uphill and upstairs movements to verify the repeatability of the proposed method after our robot is repaired.

## Conclusion

To enhance the stability of a quadruped robot with a manipulator on a sloped terrain, this study introduces a balance control method based on whole-body synergy, focusing on dynamically adjusting the robot’s overall balance through effective utilization of the manipulator’s active motion. Initially, a simplified whole-body dynamics model is developed by integrating the structural characteristics and DoF configuration of the quadruped robot with a manipulator. Enhancements to the control framework are made for improved motion tracking accuracy, incorporating system angular momentum optimization and manipulability of the manipulator into the hierarchical optimization task set to enhance manipulator and robot system controllability. Subsequently, to ensure stable walking of the quadruped robot with a manipulator on an irregular terrain, a balance control strategy centered on manipulator adaptive adjustment is proposed. This strategy establishes a mapping between manipulator motion and robot attitude while maintaining overall system balance. Real-time estimation of terrain angles guides manipulator motion planning to uphold robot balance and enhance movement efficiency. Comparative tests conducted in simulation environments and experiments demonstrate successful completion of a 25° slope walk by the quadruped robot with a manipulator under manipulator adaptive adjustment control. Results indicate reduced velocity fluctuations, smoother foot-end force, and improved stability during slope climbing, validating the efficacy and superiority of the manipulator-based adaptive adjustment strategy put forward in this research.

Looking ahead, addressing challenges related to external forces impacting quadruped robot operation during locomotion is imperative to further enhance control complexity and operational efficiency.

## Data Availability

The data used to support the findings of this study are available from the first author upon request.
